# Sperm from Hyh Mice Carrying a Point Mutation in αSNAP Have a Defect in Acrosome Reaction

**DOI:** 10.1371/journal.pone.0004963

**Published:** 2009-03-23

**Authors:** Luis Federico Bátiz, Gerardo A. De Blas, Marcela A. Michaut, Alfredo R. Ramírez, Facundo Rodríguez, Marcelo H. Ratto, Cristian Oliver, Claudia N. Tomes, Esteban M. Rodríguez, Luis S. Mayorga

**Affiliations:** 1 Instituto de Anatomía, Histología y Patología, Facultad de Medicina, Universidad Austral de Chile (UACh), Valdivia, Chile; 2 Laboratorio de Biología Celular y Molecular, Instituto de Histología y Embriología (IHEM-CONICET), Facultad de Ciencias Médicas, Universidad Nacional de Cuyo, Mendoza, Argentina; 3 Unidad de Reproducción, Instituto de Ciencia Animal, Facultad de Ciencias Veterinarias, UACh, Valdivia, Chile; University of Florida, United States of America

## Abstract

Hydrocephalus with hop gait (hyh) is a recessive inheritable disease that arose spontaneously in a mouse strain. A missense mutation in the *Napa* gene that results in the substitution of a methionine for isoleucine at position 105 (M105I) of αSNAP has been detected in these animals. αSNAP is a ubiquitous protein that plays a key role in membrane fusion and exocytosis. In this study, we found that male hyh mice with a mild phenotype produced morphologically normal and motile sperm, but had a strongly reduced fertility. When stimulated with progesterone or A23187 (a calcium ionophore), sperm from these animals had a defective acrosome reaction. It has been reported that the M105I mutation affects the expression but not the function of the protein. Consistent with an hypomorphic phenotype, the testes and epididymides of hyh mice had low amounts of the mutated protein. In contrast, sperm had αSNAP levels indistinguishable from those found in wild type cells, suggesting that the mutated protein is not fully functional for acrosomal exocytosis. Corroborating this possibility, addition of recombinant wild type αSNAP rescued exocytosis in streptolysin O-permeabilized sperm, while the mutant protein was ineffective. Moreover, addition of recombinant αSNAP. M105I inhibited acrosomal exocytosis in permeabilized human and wild type mouse sperm. We conclude that the M105I mutation affects the expression and also the function of αSNAP, and that a fully functional αSNAP is necessary for acrosomal exocytosis, a key event in fertilization.

## Introduction

Intracellular transport of macromolecules is an essential process in cell physiology. Most steps of this process require apposition and fusion of membrane-bound compartments. Soluble N-ethylmaleimide sensitive factor attachment protein α (αSNAP) is a ubiquitous protein present in all eukaryotic cells playing a key role in membrane fusion [1]. It participates in the activation of SNAP receptors (SNAREs), which are membrane associated proteins necessary for membrane fusion [2]. SNAREs localizing in the same compartment form cis SNARE complexes, which are inactive [3]. αSNAP binds to these complexes and recruits N-ethylmaleimide-sensitive factor (NSF), an ATPase that catalyzes the disruption of the complexes rendering active monomeric SNARE proteins. Activated SNAREs in the compartments that are going to fuse form trans complexes (i.e., SNAREs in one membrane bound to complementary SNAREs in the opposite membrane) bringing the two membranes in close proximity and promoting lipid mixing and membrane fusion.

Regulated secretion requires the fusion of exocytic granules with the plasma membrane and depends on αSNAP [4]–[6]. In particular we have documented that this protein is necessary for acrosomal exocytosis in human sperm [7]. The acrosome is a large membrane-limited granule that overlies the nucleus of mature sperm [8]. When in contact with the extracellular matrix surrounding the oocyte -named zona pellucida-, the spermatozoon undergoes acrosomal exocytosis. This secretory process releases a set of enzymes that facilitates the penetration of the zona pellucida and exposes membrane domains in the sperm that are important for fertilization. Interestingly, in resting sperm SNAREs are engaged in cis complexes [9]. Upon initiation of the acrosomal exocytosis, αSNAP -in association with NSF- disassembles cis SNARE complexes that can then form trans complexes and drive membrane fusion.

Many diseases have been associated to mutations in proteins involved in intracellular transport [10]. In particular, hydrocephalus with hop gait (hyh) is a recessive mouse disease that arose spontaneously in the C57BL/10J strain [11]. Affected mice exhibit dilatation of the cerebral ventricles at birth and develop hopping gait. However, heterogeneous phenotype expression has been recently described [12]. It has been shown that *Napa* -the gene encoding for αSNAP- is mutated in hyh mice [13], [14]. A G→A missense mutation in exon 4 causes the substitution of a highly conserved methionine for isoleucine at position 105 (M105I) in one of the α-helical domains of the protein.

The aim of this study was to asses whether the M105I mutation in αSNAP could affect the sperm acrosome reaction, and hence, the fertility of hyh male mice. Our results show that animals displaying a slowly progressive phenotype produce morphologically normal and motile sperm, but have strongly reduced fertility. We demonstrate that these cells have a defective acrosomal exocytosis due to a functional deficiency of αSNAP. Moreover, we show that αSNAP carrying the M105I mutation is not fully functional for the acrosome reaction.

## Results

### Male hyh mice have a strongly reduced fertility

**Figure 1:**
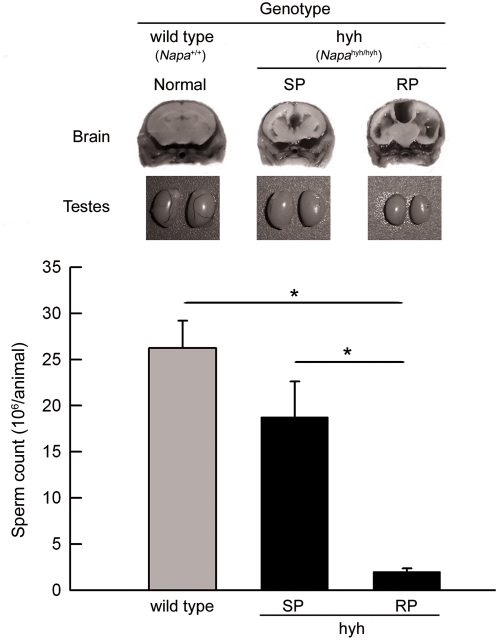
Testes phenotype and sperm count in wild type and hyh (hyh) mice. Hyh mice from the RP group developed a very severe hydrocephalus. On the other hand, mutant mice from the SP group developed a mild hydrocephalic process and less neuropathological alterations. Brains from wild type (normal phenotype), and SP hyh and RP hyh mice from the same litter (70 day old) were fixed with Bouin solution by vascular perfusion and coronally sectioned at the level of the optic chiasm. Representative images of brain and cerebral ventricles phenotypes are shown. Images of the testes from the same animals are shown below. Cauda epididymidis sperm were obtained as explain in Materials and Methods and counted. Data represent the mean±SEM from at least 6 independent experiments. * p<0.01 (one way ANOVA, Tukey's test).

We have previously described that hyh mice present a heterogenous neuropathological and clinical phenotype [12]. Seventy percent of mutant mice develop a rapidly progressive (RP) phenotype and most of them die during the first two months of life. However, 30% of them grow with a mild or slowly progressive (SP) phenotype and survive for up to 2 years [12]. The more affected animals (RP) have substantial ventricular dilatations, small testes (42.1±1.1 mg; n = 8), and strongly diminished number of sperm (Fig. 1). In contrast, SP mice have moderate ventricular dilatation and almost normal testis size (wild type, 91.6±2.3 mg, n = 20; SP, 83.1±1.8 mg, n = 16; mean±SEM) and sperm count in the cauda epididymidis (Fig. 1).

SP mice present a preserved general motor activity [12] and are able to copulate; a vaginal plug was normally observed in their female partners. However, male fertility was strongly reduced (Table 1). To rule out any problem affecting the complex processes occurring upstream of gamete interaction (i.e., mating behavior, ejaculation, etc.), fertilization was assessed in vitro (Table 2). This protocol showed a remarkable difference in fertilizing probability between sperm from wild type and SP mice. Even duplicating the concentration of sperm from hyh mice used in the assay (Table 2, experiment B) the fertilization was not as efficient as when sperm from wild type mice were used. In conclusion, the sperm from SP mice have a strongly reduced fertilizing ability.

**Table 1:** Reproductive efficiency of wild type and SP hyh mice.

Male	Female	Productive matings^a^	Litter size	Number of litters	Relative fecundity^b^
wt	wt	90.9% (13/15)	7.5	5.1	34.76
het	het	89.7% (113/126)	7.3	5.4	35.35
hyh	het	0% 0/24	0.0	0.0	0.0
hyh	hyh	1.4% (2/148)^c^	5.5	4.5	0.33

wt: wild type (*Napa^(+/+)^*); het: heterozygous (*Napa^(hyh/+)^*); hyh: mutant homozygous (*Napa^(hyh/hyh)^*) with the slow progressive phenotype.

a Matings are considered “productive” if at least one offspring was born.

b “Relative fecundity” is obtained as: (productive mating/100)×(litter size)×(number of litters); the value obtained is a measure of the overall fecundity according to the Handbook of Genetically Standardized JAX Mice [33].

c The same hyh male had two productive matings.

**Table 2:** In vitro fertilization (IVF) assays using sperm from wild type and SP hyh mice.

	sperm (sperm/ml)	Fertilized (n)	Total (n)	Fertilization (%)
Experiment A	wt (2×10^5^)	15	29	52
	hyh (2×10^5^)	5	50	10 ^**^
Experiment B	wt (2×10^5^)	27	39	69
	hyh (4×10^5^)	31	67	46 ^*^

Metaphase II eggs from wild type (*Napa* ^(+/+)^) female mice were incubated with sperm from wild type *(Napa* ^(+/+)^, wt) or mutant homozygous with the slow progressive phenotype (*Napa^(hyh/hyh)^*, hyh) mice. Following capacitation, sperm from wild type and hyh mice were diluted to 2 or 4×10^5^ sperm/ml and coincubated with eggs from wild type female mice. In experiment “A”, the same number of sperm cells from wt and hyh mice was used (2×10^5^ sperm/ml). In experiment “B” the number of sperm from hyh mice was duplicated (4×10^5^ sperm/ml). (*, p<0.02 or **, p<0.001 for hyh versus wt; Fisher's exact probability test).

### Sperm from hyh mice have a deficient acrosome reaction

**Figure 2:**
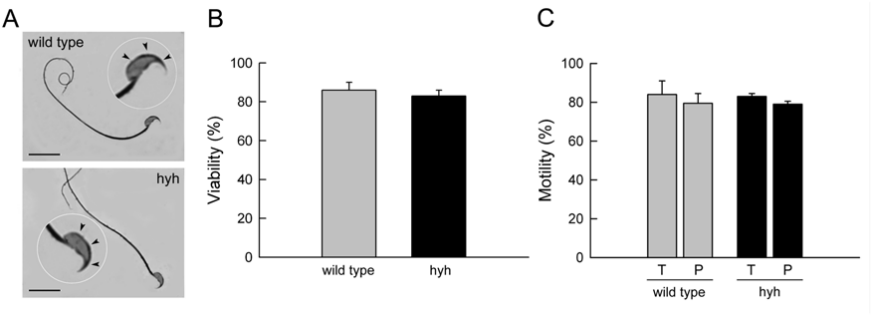
Sperm characteristics in wild type and SP hyh (hyh) mice. (A) Bright field micrographs of sperm stained with Coomassie G-250 [30] from wild type and mutant (hyh) mice showing normal cell and acrosome morphology. Scale bar, 15 µm. (B) Sperm viability for wild type and mutant (hyh) mice was assessed by Eosin Y staining. (C) The percentage of motile sperm (T) and having progressive motility (P) was assessed in wild type and mutant (hyh) mice. Data represent the mean±SEM of at least four independent experiments.

**Figure 3:**
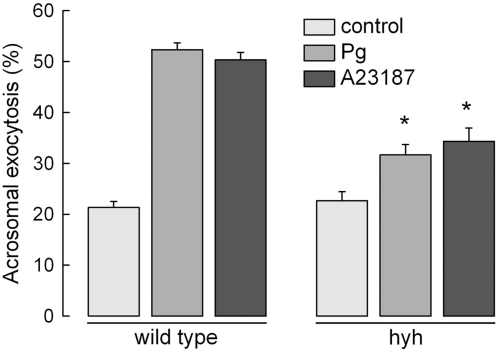
Sperm from SP hyh mice have a deficient acrosomal reaction. Sperm from wild type and SP hyh (hyh) mice were collected from the cauda epididymidis, incubated under capacitation conditions for 1 h and stimulated with buffer (control), 10 µM progesterone (Pg) or 10 µM A23187 (A23187) for 15 min at 37°C. The cells were spotted on slides and fixed in ice-cold methanol. Acrosomal status was evaluated in at least 200 sperm by staining with TRITC-PNA. The data represent the mean±SEM of three independent experiments (*, significant differences between same groups for wild type and hyh mice, P<0.001, Student's t test).

Several causes can render a spermatozoon unable to fertilize an oocyte. We analyzed the morphology, viability, and motility of SP sperm. The results summarized in Fig. 2 show that cells collected from the cauda epididymidis had a normal aspect and an apparently unaltered acrosome. They also presented a pattern of viability and motility undistinguishable from wild type sperm. Moreover, spontaneous acrosome reaction was similar in sperm from wild type and mutated mice (Fig. 3). However, when these cells were incubated under capacitating conditions and challenged with progesterone, a strongly reduced acrosome reaction was observed in sperm from mutated animals (Fig. 3). This phenomenon was observed even when acrosome reaction was induced with the calcium ionophore A23187, indicating that the defect was not in the signal transduction mechanism opening the calcium channels that triggers exocytosis but in the mechanism of exocytosis itself (Fig. 3).

### αSNAP concentration is reduced in the testis and epididymis of hyh mice, but normal in sperm from these animals

**Figure 4:**
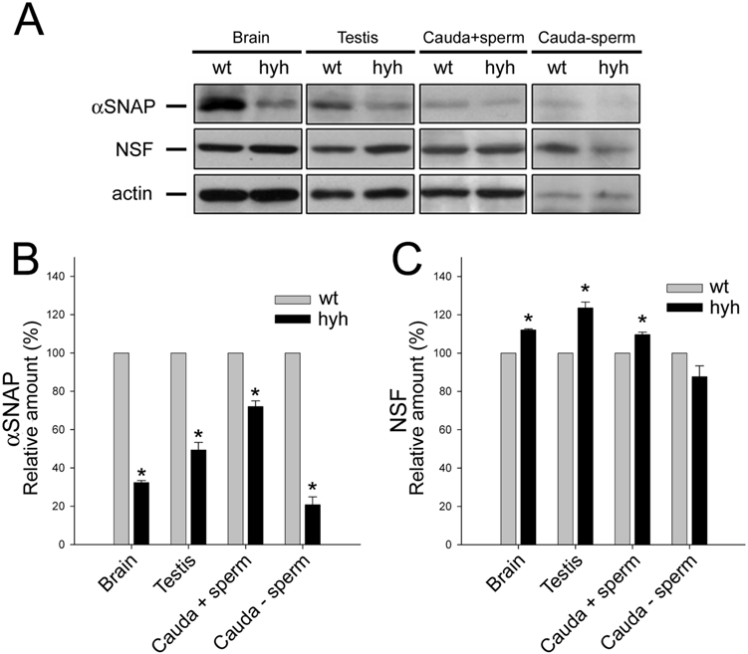
αSNAP and NSF expression in the reproductive tract of wild type (wt) and SP hyh (hyh) mice. (A) Proteins extracted from testis and cauda epididymidis of wt and SP hyh mice were analyzed by Western blot using an antibody recognizing αSNAP (upper panel) or NSF (middle panel). Signals detected with an anti-actin antibody served as internal controls for equal protein loading (lower panel). Cauda epididymidis extracts were obtained before (Cauda+sperm) and after (Cauda-sperm) sperm were washed out from the organ. Brain was used as a positive control. Blots are representative of 3 or 4 independent experiments. (B, C) Densitometric analysis of Western blot for αSNAP (B) and NSF (C). Black bars (mean±SEM, N = 3 or 4) refer to the relative amount of each protein in hyh samples compared to wt. * p<0.05 (Student's t-test).

It has been previously reported that the amount of αSNAP is strongly reduced in the brain of hyh mice, indicating that the mutation causes an alteration in the steady state balance of this protein [13], [14]. As the expression of αSNAP protein has not been previously described in mouse male reproductive tract (except in spermatogenic cells, [15], we decided to investigate the presence and distribution of this protein in testis, epididymis and sperm of wild type and SP mice. The hypothesis was that the abnormal acrosome reaction observed in sperm from SP mice is caused by low levels of αSNAP in these cells. As it has been described in the brain, the Western blot analysis revealed that the amount of αSNAP was strongly reduced in testes from SP mice. Similarly, decreased levels of αSNAP were found in the epididymis of SP mice, a finding that was more evident when sperm were washed out from the epididymal lumen (Fig. 4A and 4B). We also evaluated the expression of NSF, the ATPase that works in association with αSNAP to disassemble SNARE complexes. Interestingly, the amount of NSF was not decreased in testes and epididymides of SP mice; on the contrary, a small but consistent increase was observed (Fig. 4A and 4C).

**Figure 5:**
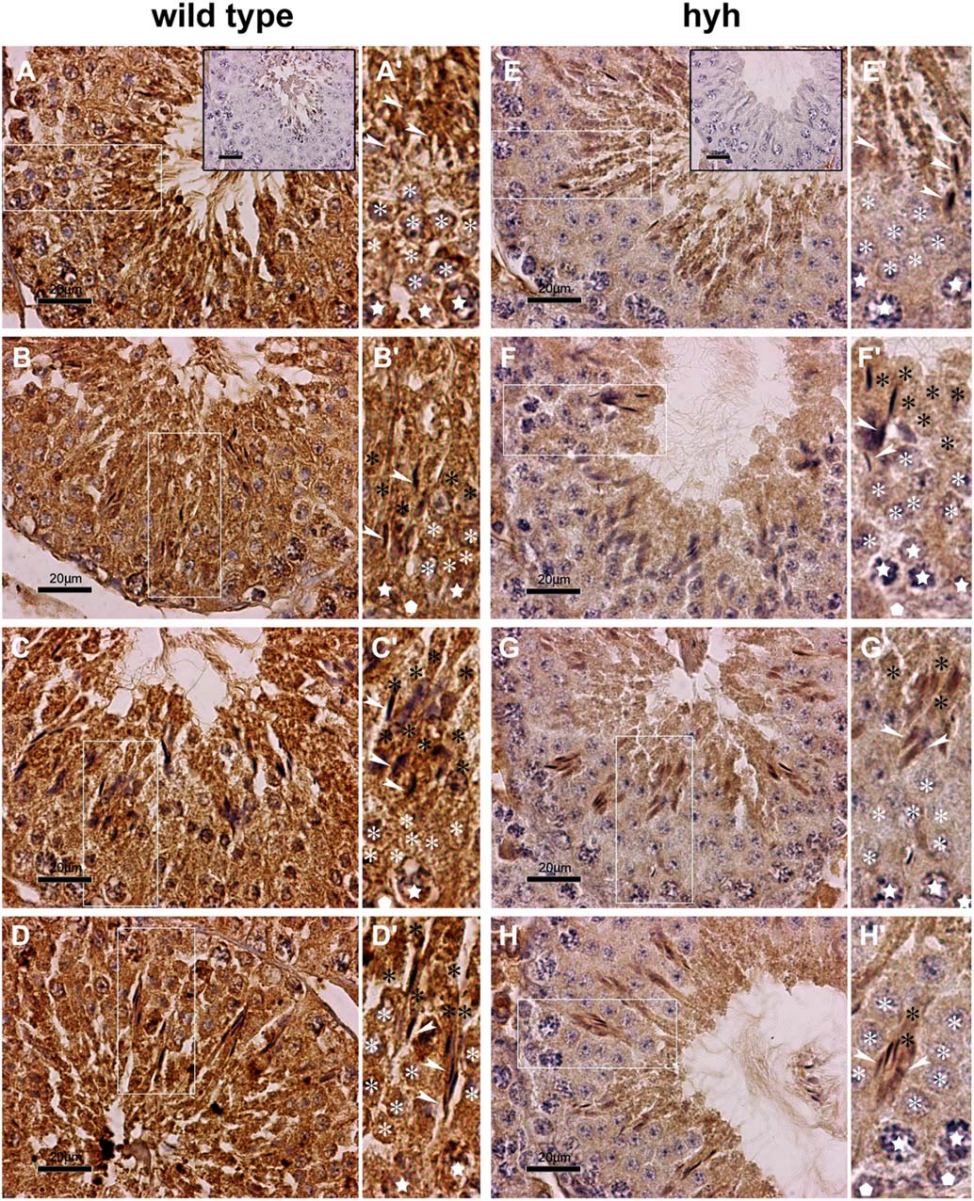
Immunolocalization of αSNAP in seminiferous epithelium. Light micrographs of testis sections from wild type (A–D) and SP hyh mice (E–H) showing general and specific patterns of immunoperoxidase staining using an αSNAP-specific antibody. Background staining with hematoxylin. Different stages of epithelial maturation cycle in normal and mutant testis are presented in comparative mode. A'–H': High magnification images of the regions boxed in the corresponding panel (A–H). Epithelial polarization is oriented upwards. The symbols used are: spermatogonium (white pentagon); primary spermatocytes (white stars); round spermatids (white asterisk); elongated spermatids with high polarization, indicating the residual body or axonemal region (black asterisk) and the heads (white arrowheads). Controls without primary antibodies are shown in the inserts (A and E). In wild type animals, a strong immunoreaction was observed in the whole ephitelium. In contrast, mutant mice showed a notably diminished immunoreaction compared to that of wild type mice (compare E–H to A–D panels). This difference was not evident in spermatids undergoing elongation process. Scale bar, 20 µm.

**Figure 6:**
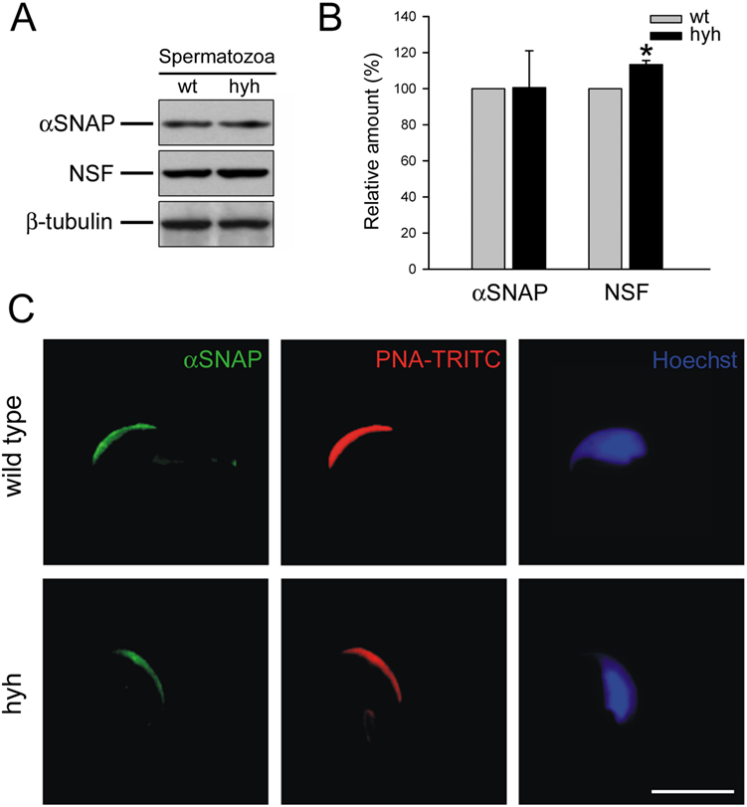
αSNAP expression and localization in sperm from wild type (wt) and SP hyh (hyh) mice. (A) Sperm protein extracts obtained from wt and hyh mice were analyzed by Western blot using an antibody recognizing αSNAP (upper panel) or NSF (middle panel). Signals detected with an anti β-tubulin antibody served as internal controls for equal protein loading (lower panel). Blots are representative of seven independent experiments. (B) Densitometric analysis of Western blot for αSNAP and NSF. Black bars (mean±SEM, N = 7) refer to the relative amount of each protein in hyh samples compared to wt (gray bars). * p<0.05 (Student's t-test). (C) αSNAP localizes to the acrosomal region in mouse spermatozoa. Sperm from wild type and hyh mice were fixed, permeabilized and triple-stained with an anti-α/βSNAP antibody (green); TRITC-PNA, a lectin that recognizes the intra-acrosomal content (red); and Hoechst 33258 to visualize the nucleus of the cell (blue). Shown are epifluorescence micrographs of typically stained cells. Scale bar, 10 µm.

Supporting the results obtained by Western blot, a strong difference in αSNAP immunostaining was observed in the seminiferous epithelium from wild type and SP animals (Figure 5). This difference was evident in pre- and post-meiotic cellular stages, including spermatogonia, primary spermatocytes and round spermatids. However, the immunoreactive pattern of elongated spermatids in mutant animals was very similar to that found in wild type mice. To evaluate if the immunostaining pattern observed in elongated spermatids was maintained in mature sperm, the amount of αSNAP was assessed by Western blot in spermatozoa obtained from cauda epididimydis. The results showed that the αSNAP protein level in sperm from SP mice was identical to that found in wild type cells, and that NSF was -as in the other tissues- slightly increased (Fig. 6A and 6B). Immunofluorescence for αSNAP also showed a similar pattern in sperm from wild type and SP mice (Fig. 6C). A distinct acrosomal labeling was observed (Fig. 6C), which disappeared when the antibody was absorbed with recombinant αSNAP (data not shown). In conclusion, the testis and epididymis of SP mice present a reduced concentration of αSNAP. In contrast, the amount and distribution of this protein is normal in mature sperm from SP mice.

### Wild type αSNAP rescues acrosomal exocytosis in sperm from hyh mice

**Figure 7:**
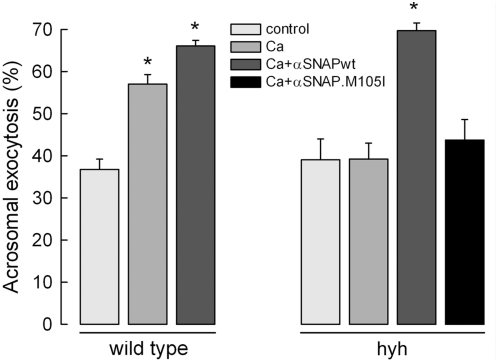
αSNAP rescues acrosomal exocytosis in sperm from SP hyh mice. Sperm from wild type and SP hyh mice were collected from the cauda epididymidis and incubated under capacitation conditions for 1 h. The cells were then permeabilized and stimulated with buffer (control) or 0.5 mM CaCl_2_ (10 µM free Ca^2+^). When indicated, 15 nM recombinant wild type αSNAP (αSNAPwt) or the M105I mutant (αSNAP.M105I) were added to the assay. The samples were incubated for 15 min at 37°C. At the end of the incubation, the cells were spotted on slides and fixed in ice-cold methanol. Acrosomal status was evaluated in at least 200 sperm by staining with TRITC-PNA. The data represent the mean±SEM of three independent experiments (*, significant differences with respect to control for wild type and hyh mice, P<0.01, one way ANOVA, Dunnett's test).

The above results indicate that the acrosomal exocytosis defect in hyh mice was not due to a diminished amount of αSNAP. A possible explanation for this observation is that the low concentrations of αSNAP in the testis and epididymis alter the normal maturation of the exocytic machinery of the sperm. Hence, even with a normal quantity of the protein, the sperm may be unable to exocytose. To test this hypothesis, we assessed whether we could restore exocytosis by adding wild type αSNAP to sperm from SP mice. To this end, we set up a plasma membrane-permeabilized mouse sperm model similar to the one we routinely use for human sperm [16], [17]. The percentage of permeabilized cells without acrosome under resting conditions was 16% higher than that found in untreated cells (compare Fig. 7 with Fig. 3), suggesting that some sperm may have lost their acrosome during the permeabilization procedure. However, upon stimulation with calcium, an additional 18% of the cells reacted (Fig. 7), indicating that the treatment did not inactivate the exocytic machinery. A similar percentage of calcium induced exocytosis in permeabilized sperm has been previously reported by us [16] and by other authors [18]. In contrast, sperm from mutated mice did not respond to calcium stimulation, in agreement with the results obtained with A23187 in intact cells (compare Fig. 7 with Fig 3). When wild type αSNAP was added to the assay, exocytosis was completely recovered, suggesting that the primary defect in these cells is a deficient amount of functional αSNAP and not an abnormal maturation of sperm in SP mice (Fig. 7). However, the previous Western blot analysis and immunofluorescence images showed that these cells have normal levels of αSNAP, suggesting that the M105I mutation affects directly the function of the protein. To test this possibility, a recombinant αSNAP.M105I protein was produced, purified, and added to permeabilized sperm from SP mice. The mutated protein was unable to rescue acrosomal exocytosis in these cells at 15 nM (Fig. 7), although a partial recovery was observed at 60 nM (data not shown). In conclusion, although carrying normal levels of αSNAP, hyh sperm cells have a deficient acrosome reaction because the mutated protein cannot support exocytosis with the same efficiency than the wild type molecule

### αSNAP.M105I inhibits exocytosis in sperm from wild type mice and normal human donors

**Figure 8:**
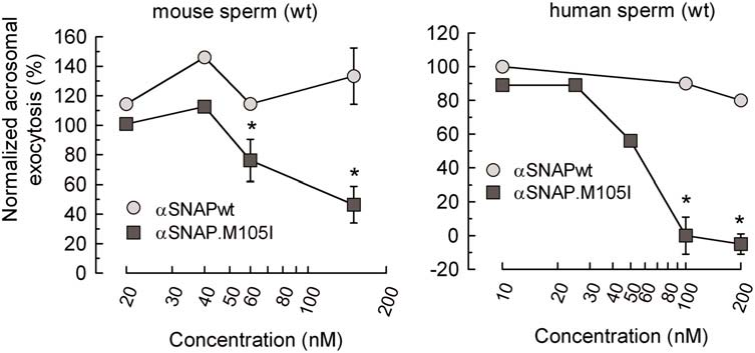
Inhibitory effect of αSNAP.M105I on the acrosomal exocytosis of permeabilized normal mouse and human sperm. (A) Sperm from wild type mice were collected from the cauda epididymidis and incubated under capacitating conditions for 1 h. (B) Human sperm were collected from ejaculates as previously described [9]. Mouse and human cells were then permeabilized and stimulated with 10 µM free Ca^2+^ in the presence of different concentrations of wild type αSNAP (αSNAPwt) or the M105I mutant (αSNAP.M105I) for 15 min at 37°C. At the end of the incubation, the cells were spotted on slides and fixed in ice-cold methanol. Acrosomal status was evaluated in at least 200 sperm by staining with TRITC-PNA (mouse sperm) or FITC-PSA (human sperm). For each experiment, acrosomal exocytosis was normalized by subtracting the number of reacted spermatozoa in the non-stimulated samples (mean±SEM: 44.8%±4.3% and 11.7%±1.2%, for mouse and human sperm, respectively) from all values, and expressing the resulting values as a percentage of the acrosome reaction observed in cells stimulated with 10 µM free Ca^2+^ in the absence of added αSNAP (63.4%±3.7% and 23.7%±1.2%, for mouse and human sperm, respectively). The data represent the mean±SEM of three independent experiments. (*, significant differences with respect to wild type αSNAP, P<0.01, Student's t test).

Although αSNAP is a required factor for intracellular transport, in some experimental models -including the acrosomal exocytosis- an excess of the wild type protein inhibits membrane fusion [7], [19]–[21]. Hence, we tested the effect of wild type and mutated proteins in the acrosomal exocytosis assay using permeabilized sperm from wild type mice. The results showed that M105I mutant had a dose-dependent inhibitory effect on exocytosis, whereas, in the same range of concentrations, the wild type protein could even improve secretion (Fig. 8A). To assess the effect of the M105I mutant in another model, the protein was added to permeabilized human sperm. In the range of concentration tested, wild type αSNAP did not affect secretion; in contrast, the M105I mutant was strongly inhibitory (Fig. 8B). None of the recombinant proteins affected basal exocytosis in the absence of calcium in both systems (data not shown). In conclusion, αSNAP.M105I, as compared with the wild type protein, has a reduced ability to restore exocytosis and an enhanced potency to inhibit the process, indicating that this mutation alters the functionality of the protein.

## Discussion

The acrosome reaction is a special type of regulated secretion [22]. At fertilization, it is initiated by a complex signal transduction pathway triggered by the contact of the sperm membrane with components of the zona pellucida [8]. At the end of this pathway, cytoplasmic calcium increases and activates the membrane fusion machinery that opens hundred of pores connecting the acrosomal lumen with the extracellular medium. The expansion of these pores promotes the fenestration of the membrane and the formation of hybrid vesicles. Although with particular characteristic, acrosome exocytosis is a regulated secretion sharing the same basic mechanism of membrane fusion that has been described in other secreting cells, such as neurons and endocrine cells [22]. Moreover, the special features of the acrosomal exocytosis have proven useful to study several aspects of the secretion process that are more difficult to assess in other cells [23].

Acrosome reaction is a required event to achieve fertilization in mammals [8]. Enzymes present in the acrosomal granule must be released to facilitate sperm penetration through the zona pellucida. Hence, it would be expected that malfunctions in factors necessary for secretion would affect the fertilizing potential of sperm [24], [25]. However, since secretion is a key cellular function necessary for many survival mechanisms, most mutations in these factors render animals unable to survive to reproductive age. Interestingly, when they do survive, they are in many cases fertile, suggesting that compensatory mechanisms may overcome the malfunction or absence of some factors [26]. Hyh mouse harboring a point mutation in the *Napa* gen coding for αSNAP is the first example of a fertility problem directly related to a factor necessary for the general mechanism of regulated secretion that is specifically affecting the acrosome reaction. Mutant mice with a mild clinical and neuropathological phenotype are able to copulate and the amount of live and motile sperm that they produce is similar to that of wild type mice; however, they have a much reduced fertility. Even when fertilization was assessed in vitro, sperm from hyh mice behave poorly as compared with sperm from wild type mice. Notwithstanding, the difference between genotypes was less dramatic in the in vitro assays than in the mating studies. Thus, other defects not assessed by in vitro fertilization might contribute to this phenotype. In this context, although SP mice present a preserved general motor activity and are able to copulate, they do have gait and equilibrium impairments [12] that could affect the frequency and efficiency of copulation.

Acrosome reaction experiments indicate that the basic problem in sperm from hyh mice is a reduced capacity to undergo exocytosis upon stimulation with progesterone and even with a calcium ionophore. The fact that exogenous αSNAP can rescue acrosomal exocytosis in these cells is a strong evidence that the principal defect is a malfunction in the endogenous protein. Worth noticing is that the M105I mutation is in a region of αSNAP that does not interact with the SNARE complex [27]. Moreover, the protein can bind to and disassemble SNARE complexes in vitro in a way similar to the wild type protein [13], [14]. These observations, together with the fact that the hyh mouse presents a diminished amount of αSNAP in brain, have lead to the conclusion that the alteration in brain development in these animals is principally due to an insufficient amount of protein and not to a malfunction of the mutated molecule [13]. However, a functional defect of the mutated protein has never been ruled out [13], [14]. Our observations in sperm point to a different mechanism. We confirmed that the mutated protein has an altered steady state distribution in several tissues; the levels of αSNAP in testis and epididymis were significantly lower in the mutated animals. In contrast, we found normal amount of this protein in sperm. Therefore, it was unlikely that the defect in acrosomal exocytosis was due to a decreased amount of αSNAP. These observations suggested that the mutated protein has some intrinsic malfunction. This was confirmed by the fact that the mutated protein was less effective in restoring exocytosis than the wild type protein when added to permeabilized sperm from hyh mouse. Moreover, the mutated protein was inhibitory when added to normal mouse and human sperm. It is worth noticing that an excess of wild type αSNAP is also inhibitory, but at much higher concentrations [7]. Our results indicate that the M105I mutation alters the normal steady state balance of αSNAP and also affects its function. The protein may bind SNARE complexes and may promote their disassembly as the wild type protein [13], [14], but in the complexity of a cellular environment with several other interacting factors, the mutant behaves differently than the wild type protein in the acrosomal secretory processes. In fact, co-immunoprecipitation studies using brain lysates suggest that the M105I mutation may affect the αSNAP/SNARE complex interaction [14]. Relatively high concentrations of NSF -the other key factor for SNARE complex disassembly- were found in testis, epididymis, and sperm of hyh mice, likely as a compensatory response to the diminished amount of functional αSNAP in the cells.

The deletion of αSNAP is embryonically lethal in mice [13]; hence the M105I mutant must conserve a certain degree of functionality for intracellular transport and regulated exocytosis, but cannot support acrosomal exocytosis. αSNAP has a well characterized function in SNARE complex disassembly. In sperm it is necessary for the activation of SNARE proteins upon sperm stimulation [7]. However, it may have other still not well characterized functions [21] that may be altered in the M105I mutant. The methionine in the 105 position is well preserved along evolution. This residue is in the convex surface of the protein, opposite to the one supposed to bind SNARE complexes; however, mutation analyses indicate that this area is also important for αSNAP function [27]. All in all, the molecular mechanism for the mutant malfunction remains to be determined.

## Materials and Methods

### Ethics Statement

Handling, care and processing of animals were carried out according to the regulations approved by the Bioethics Committee of the Universidad Austral de Chile and the Council of the American Physiological Society. The protocol for handling human sperm samples has been approved by the Bioethics Committee of the Medical School of the Universidad Nacional de Cuyo, Argentina.

### Reagents

Recombinant streptolysin O (SLO) was obtained from Dr Bhakdi (University of Mainz, Mainz, Germany). Tetramethylrhodamine isothiocyanate-labeled peanut agglutinin (TRITC-PNA) and VECTASTAIN Universal Elite ABC kit (PK-6200) were from Vector Laboratories Ltd. (Peterborough, UK). Fluorescein isothiocyanate-coupled *Pisum sativum* (FITC-PSA) was from Sigma Chemical Co. (St. Louis, Missouri, USA). Hoechst 33258, Sytox Green, A23187, and Alexa Fluor 488-conjugated goat anti-mouse immunoglobulin G were from Molecular Probes (Eugene, OR, USA). Mouse monoclonal anti-α-SNAP (clone 4E4) and anti-α/β-SNAP (purified IgG, clone 77.1) antibodies were purchased from Exalpha Biologicals (Maynard, MA, USA) and Synaptic Systems (Göttingen, Germany), respectively. Mouse anti-NSF monoclonal antibody (clone 7) was purchased from BD Biosciences Pharmingen. Monoclonal mouse anti-actin antibody (JLA20) was obtained from the Developmental Studies Hybridoma Bank (Iowa City, IA, USA) and monoclonal mouse anti-β-tubulin (clone 2-28-33) was from Sigma Chemical Co. All other chemicals were analytical-grade and were purchased from Sigma Chemical Co. or ICN Biochemicals (Aurora, Ohio, USA).

### Recombinant proteins

Plasmid pQE9 encoding wild type αSNAP was a kind gift from Dr S.W. Whiteheart (University of Kentucky, Lexington, KY, USA). Plasmid pET28 encoding αSNAP.M105I was a generous gift from Dr. Phillys Hanson (Washington University, St. Louis, Missouri, USA). Recombinant His_6_-tagged αSNAP wild type and αSNAP.M105I were purified from the cytosolic fraction of Escherichia coli XL-1 blue (Stratagene, Cambridge, UK) on Ni-NTA-agarose based on previously published methods [7].

### Animals

Mice were obtained from The Jackson Laboratory (Bar Harbor, ME, USA), where the *hyh* mutation originally arose in the C57BL/10J inbred strain and was subsequently placed on a B6C3Fe-*a/a* (C57BL/6J female X C3HeB/FeJ-*a/a* male) hybrid background [11]. These animals were bred into a colony at Facultad de Medicina, Universidad Austral de Chile, Valdivia, Chile. Eight different maternal sub-lines have been developed [12]. This study included only adult animals (8–20 weeks) from different sub-lines and generations. Mice were fed *ad libitum* with rodent food and kept under a constant photoperiod of light/dark 12∶12 h and room temperature of 22°C.

### Mice mating system

Mice (8–12 week old) were genotyped and mated using the monogamous mating system, except in the case of mating homozygous mutant male with homozygous mutant female were polygamous mating was utilized (one male with up to three females). Vaginal plug was used as an indication that mating had occurred. If no litters were produced after 10 weeks the mating was considered non-productive.

### Gamete collection

Metaphase II eggs were obtained from 6 to 8 week old wild type female mice superovulated by injection of 5 IU of pregnant mare serum gonadotrophin (PMSG; Syntex S.A., Argentina) followed 48 h later by 5 IU of human chorionic gonadotrophin (hCG; Syntex S.A., Argentina). Eggs were collected in Whitten's medium [28] containing 0.4% (w/v) polyvinylpyrrolidone (PVP) and 20 mM Hepes buffer at pH 7.4. Cumulus cells were removed using 0.3% hyaluronidase for 1–3 min in the same solution. Eggs were collected and placed immediately into the fertilization drop for in vitro fertilization. Mouse sperm were collected from 12–24 week old wild type and hyh male mice. Both cauda epididymides from a single male were placed in a 900 µl drop of human tubal fluid (HTF) medium [29] supplemented with 0.5% BSA. Several incisions were made in the tissue and the sperm allowed swimming into the medium for 20 min at 37°C. The resultant cell suspension was counted, adjusted to 7–10×10^6^ sperm/ml, and incubated for 1 h at 37°C in an atmosphere of 5% CO_2_/95% air. Human semen samples were obtained from normal healthy donors. Highly motile sperm were recovered following a swim-up separation in HTF supplemented with 0.5% BSA for 1 h at 37°C in an atmosphere of 5% CO_2_/95% air. Concentration was adjusted to 5–10×10^6^/ml, and incubation proceeded for at least 2 h.

### In vitro fertilization

All IVF procedures were performed approximately 14 hours post hCG injection using HTF medium. Metaphase II eggs were obtained from 5–6 wild type female mice. Only eggs with visible polar body were collected and separated in two groups. Sperm from two wild type male mice and from at least two hyh male mice were obtained as explained above. Following capacitation, sperm from wild type and hyh mice were diluted to 2 or 4×10^5^ sperm/ml. Eggs and sperm were mixed in a 500 µl fertilization drop of HTF medium containing 0.5% BSA, and then cultured at 37°C in an atmosphere of 5% CO_2_/95% air. After 3 hours, eggs were washed six times to remove any loosely attached sperm, and then cultured for 5 h. Finally, embryos were fixed in 3.7% paraformaldehyde in PBS for 15 min, permeabilized with 0.1% Triton X-100 and stained with 10 µM Sytox Green (Molecular Probes) for DNA staining. Fertilization was evaluated by the presence of the second polar body and the formation of both the male and female pronuclei by epifluorescence microscopy.

### Evaluation of mouse sperm morphology, viability and motility

Wild type and mutant sperm were collected as explained and adjusted to a concentration of 5–10×10^6^ cells/ml in HTF. To determine morphology of sperm and acrosome, a 5 µl aliquot was stained with Coomassie G-250 [30] and observed under 100× oil immersion lens. To assess sperm plasmalemma integrity (i.e., viability), 10 µl of sperm suspension and 1 µl of Eosin-Y solution (5 g/l) were mixed, and the percentage of stained cells was determined by counting 200 sperm (bright-field microscopy, 100× oil immersion lens). To assess motility in a sample, at least 200 sperm in two independent aliquots were classified as having progressive or non-progressive motility, or being immotile (bright-field microscopy, 40×).

### Immunohistochemistry

Testes were rapidly removed from hyh and wild type mice and fixed in Bouin fixative solution for 48 h at room temperature. Paraffin sections were processed for immunohistochemistry as previously described [7] using mouse monoclonal anti-α-SNAP (clone 4E4) as primary antibody. Omission of the incubation in the primary antibody was used as a control of the immunoreaction. Sections were examined under a Zeiss Axioskop microscope equipped with a digital camera (Nikon CoolPix 5000).

### Indirect Immunofluorescence

Sperm suspensions from hyh and wild type mice were fixed in 2% paraformaldehyde. Indirect immunofluorescence was performed as described [9] using the mouse monoclonal anti-α/β-SNAP antibody. Coverslips were mounted in Gelvatol, and examined under a Zeiss Axioskop microscope equipped with epifluorescence optics and a digital camera (Nikon Coolpix 5000). Background was subtracted and brightness/contrast were adjusted in the images to render an all or nothing labelling pattern using Corel Draw version 12 (Corel, Ottawa, Ontario, Canada).

### Protein extraction from tissue samples and sperm

Proteins from hyh and wild type mice brain, testis and epidydimis were extracted by homogenizing in a modified RIPA buffer (50 mM Tris-HCl, pH 7.4, 150 mM NaCl, 1% Triton-X-100, 1% sodium deoxycholate, 0.1% SDS) with a protease inhibitor cocktail (P2714, Sigma Chemical Co.) plus 1 mM phenylmethanesulphonylfluoride (PMSF). The homogenates were centrifuged at 6000×g for 10 minutes at 4°C to sediment unbroken cells and nuclei. Supernatants were stored at −80°C until used. Sperm proteins were isolated according to [31].

### SDS-PAGE and immunoblotting

For Western blotting, 2% (v/v) 2-mercaptoethanol was added to the samples. After boiling for 2 min, 15 µg (brain, testis, epidydimis) or 30 µg (sperm) of total protein were loaded on 10% polyacrylamide gels according to [32]. The proteins were then transferred to polyvinylidene difluoride (PVDF) membranes (Millipore, Bedford, MA). Blots were incubated for 1–2 h at 37°C with the primary antibodies diluted in PBS containing 0.05% Tween 20 and 1% BSA (anti-α-SNAP, 1∶1000; anti-NSF, 1∶5000). Horseradish peroxidase conjugated goat anti-mouse-IgG was used as secondary antibody (0.25 µg/ml) with 30 min incubations. Excess antibodies were removed by washing 5×5 min in 1× PBS. Detection was accomplished with an enhanced chemiluminescence system (SuperSignal West Pico Chemiluminescent Substrate, Pierce, Rockford, IL) and subsequent exposure to Kodak XAR film (Eastman Kodak, Rochester, NY) for 5–30 s. In order to confirm equal loading of proteins, blots were stripped and re-probed with an antibody against actin (1∶1000) or β-tubulin (1∶1000).

### Acrosome reaction in intact and permeabilized human and mouse sperm

Motile sperm were obtained as explained above. In some experiments, spermatozoa without further treatment were used to study acrosome reaction. Permeabilization of human and mouse sperm was accomplished as previously described [17]. Briefly, washed spermatozoa were resuspended in cold PBS containing 0.4–1 units/ml SLO for 15 min at 4°C. Cells were washed once with PBS and resuspended in ice-cold sucrose buffer (250 mM sucrose, 0.5 mM EGTA, 20 mM Hepes-K, pH 7.0) containing 2 mM dithiothreitol. Permeabilized sperm were incubated, when indicated, with the recombinant proteins for 15 min at 37°C before adding the exocytosis stimulators. After stimulation of intact or permeabilized sperm, incubation proceeded at 37°C for 15 min. Acrosomal status was evaluated by staining with TRITC-PNA (mouse sperm) or FITC-PSA (human sperm). At least 200 cells were scored using a Zeiss Axioskop microscope equipped with epifluorescence optics.
